# AIM‐AF: A Physician Survey in the United States and Europe

**DOI:** 10.1161/JAHA.121.023838

**Published:** 2022-03-04

**Authors:** A. John Camm, Carina Blomström‐Lundqvist, Giuseppe Boriani, Andreas Goette, Peter R. Kowey, Jose L. Merino, Jonathan P. Piccini, Sanjeev Saksena, James A. Reiffel

**Affiliations:** ^1^ St George’s University London UK; ^2^ Department of Medical Science & Cardiology Uppsala University Uppsala Sweden; ^3^ University of Modena & Reggio Emilia Modena Italy; ^4^ Department of Cardiology and Intensive Care Medicine St Vincenz Hospital Paderborn Paderborn Germany; ^5^ Sidney Kimmel Medical College at Thomas Jefferson University Philadelphia PA; ^6^ Lankenau Heart Institute Philadelphia PA; ^7^ La Paz University Hospital, Idipaz, Autonoma University Madrid Spain; ^8^ Duke Clinical Research Institute Duke University Durham NC; ^9^ Rutgers Robert Wood Johnson Medical School Piscataway NJ; ^10^ Columbia University New York NY

**Keywords:** atrial fibrillation, antiarrhythmic drug, physician, Atrial Fibrillation

## Abstract

**Background:**

Guideline recommendations are the accepted reference for selection of therapies for rhythm control of atrial fibrillation (AF). This study was designed to understand physicians’ treatment practices and adherence to guidelines.

**Methods and Results:**

The AIM‐AF (Antiarrhythmic Medication for Atrial Fibrillation) study was an online survey of clinical cardiologists and electrophysiologists that was conducted in the United States and Europe (N=629). Respondents actively treated ≥30 patients with AF who received drug therapy, and had received or were referred for ablation every 3 months. The survey comprised 96 questions on physician demographics, AF types, and treatment practices. Overall, 54% of respondents considered guidelines to be the most important nonpatient factor influencing treatment choice. Across most queried comorbidities, amiodarone was selected by 60% to 80% of respondents. Other nonadherent usage included sotalol by 21% in patients with renal impairment; dofetilide initiation (16%, United States only) outside of hospital; class Ic agents by 6% in coronary artery disease; and dronedarone by 8% in patients with heart failure with reduced ejection fraction. Additionally, rhythm control strategies were frequently chosen in asymptomatic AF (antiarrhythmic drugs [AADs], 35%; ablation, 8%) and subclinical AF (AADs, 38%; ablation, 13%). Despite guideline algorithms emphasizing safety first, efficacy (48%) was selected as the most important consideration for AAD choice, followed by safety (34%).

**Conclusions:**

Despite surveyed clinicians recognizing the importance of guidelines, nonadherence was frequently observed. While deviation may be reasonable in selected patients, in general, nonadherence has the potential to compromise patient safety. These findings highlight an underappreciation of the safe use of AADs, emphasizing the need for interventions to support optimal AAD selection.

**Nonstandard Abbreviations and Acronyms**
AFFIRM: Atrial Fibrillation Follow‐up Investigation of Rhythm Management
AIM‐AF: Antiarrhythmic Medication for Atrial Fibrillation
GWTG‐AFib: Get With The Guidelines – Atrial Fibrillation
HFrEF: heart failure with reduced ejection fraction
ORBIT‐AF: Outcomes Registry for Better Informed Treatment of Atrial Fibrillation
SHD: structural heart disease

**Clinical Perspective**

**What Is New?**

- This survey extensively explored treatment practices and attitudes toward antiarrhythmic therapies for atrial fibrillation among cardiologists and electrophysiologists in the United States and Europe.
- Despite 97% of respondents reporting that they follow treatment guidelines, there was a high level of deviation, of varying degrees, from recommendations in the 2020 European Society of Cardiology and 2014/2019 American Heart Association/American College of Cardiology/Heart Rhythm Society guidelines.

**What Are the Clinical Implications?**

- While deviations from guidelines may be reasonable in select clinical circumstances, a high degree of nonadherence raises concerns regarding patient safety.
- As the clinical presentations of AF evolve over time, and guidelines are regularly updated in line with new evidence, important safety questions arise over the extent to which physicians are keeping abreast of such updates, particularly with regard to antiarrhythmic drug use.

Atrial fibrillation (AF) is the most common sustained tachyarrhythmia and is associated with a 5‐fold increased relative risk of stroke,^1^ a 3‐fold increased relative risk of heart failure (HF),^2^ and a doubled relative risk of mortality.^3^ The prevalence of AF is increasing worldwide, predicted to affect 6 to 12 million people in the United States by 2050 and 18 million people in Europe by 2060.^4^

For the past 2 decades, the European Society of Cardiology (ESC)^5^ and the American Heart Association/American College of Cardiology/Heart Rhythm Society (AHA/ACC/HRS)^6^, ^7^ have provided physicians with guidelines to direct the management of patients with AF. Both recommend the use of antiarrhythmic drugs (AADs) for rhythm control in patients with symptomatic AF only.^5^, ^6^, ^7^ Additionally, both guidelines indicate that selection of antiarrhythmic therapies should consider arrhythmia burden, presence of underlying heart disease, severity of symptoms, and risk of side effects.^5^, ^6^, ^7^

Since the publication of the Etude en Activité Libérale de la Fibrillation Auriculaire study over 2 decades ago,^8^ the clinical landscape of AF treatment has evolved considerably, as have the guidelines. Two new AADs, dofetilide and dronedarone, are now available (although dofetilide is available in the United States only^9^), and the use of ablation therapy has become increasingly widespread. Real‐world data from the United States indicated that between 1990 and 2005, there was a 13‐fold increase in the proportion of patients with AF who received ablation (*P* < 0.001)^10^; a 12.5% annual rate of increase worldwide is predicted from 2019 to 2025.^11^ However, in the current clinical landscape, prescribing practices of physicians and their attitudes toward the management of patients with AF are poorly understood. Accordingly, the AIM‐AF (Antiarrhythmic Interventions for Managing Atrial Fibrillation) study was designed to explore cardiologist and electrophysiologist antiarrhythmic treatment practices in patients with AF. The results of the study are reported in the context of the 2020 ESC guidelines,^5^ and the 2014 and 2019 AHA/ACC/HRS guidelines.^6^, ^7^

## METHODS

Qualified researchers may request access to data including the study summary, study questionnaire with any amendments, and data set specifications for validation purposes. Only fully anonymized data will be provided.

### Study Design

The AIM‐AF study was an exploratory, online physician survey, designed by a steering committee of 9 global experts in AF. Practicing physicians from the M3 Global International Market Research Panel were invited to complete the survey, with a geographical spread across the United States and European countries involved, to avoid potential selection bias. Ethics approval was obtained from the Western Institutional Review Board committee (Ref: 1‐1337237‐1), and from the local ethics committee in Uppsala, Sweden; participants provided informed consent in accordance with institutional guidelines.

### Study Population

The survey aimed to recruit 600 clinical cardiologists, including clinical electrophysiologists and interventional electrophysiologists from the United States, Germany, Italy, Sweden, and the United Kingdom. These countries were selected to ensure that physicians from Central, Northern, Southern, and Western Europe were represented. Inclusion criteria were as follows: qualification in their specialty for >3 years and <40 years; >40% of their time spent actively treating patients; ≥30 new or existing patients with AF seen within a 3‐month period (before the COVID‐19 pandemic); and management of patients with AF who have received ablation or have been referred for ablation.

### Data Collection and Analysis

The survey was conducted between October 2, 2020, and February 12, 2021, and was intended to take 60 minutes to complete. Respondents were asked to complete 96 questions (Table S1), including a set of screening questions to ascertain demographics and eligibility. Questions were grouped on the basis of topics such as physician setting and patient caseload; treatment journey, with a focus on oral AADs; prescribing/treatment practices; and use/referral of ablation. The survey predominantly comprised closed questions, with a small number of open‐ended questions to probe physician perceptions and behaviors. Survey questions were designed to understand physicians’ general approaches to the management of patients with AF.

The survey was performed in compliance with the European Pharmaceutical Market Research Association code of conduct and in full accordance with the US Health Insurance Portability and Accountability Act of 1996.

Since no formal hypothesis was tested, data analyses are descriptive in nature. Univariate tests were conducted for comparisons between the 2 groups (United States versus Europe) and the *Z* test was applied to determine statistical significance (*P* value boundary of <0.05); however, *P* values should be considered notional since no adjustment was made for multiple testing.

To distinguish the degree of nonadherence between survey responses and recommendations from the 2020 ESC^5^ and 2014/2019 AHA/ACC/HRS guidelines,^6^, ^7^ we established 4 definitions to describe adherence: compliance with guidelines (AAD use aligns with guideline recommendations); noncompliance with guidelines (AAD use contradicts guideline recommendations); deviation from guidelines (guidelines recommend use of an alternative therapy or alternative practice in this setting); and potential noncompliance with guidelines (use in this setting *could* contradict guideline recommendations, but clinical thresholds differed between the survey questions and the guidelines, preventing absolute certainty). Estimated percentage of nonadherence was calculated for each AAD, which described the proportion of physicians who selected an AAD in at least 1 clinical circumstance that fell into any of the “noncompliance with guidelines,” “deviation from guidelines,” or “potential noncompliance with guidelines” definitions.

## RESULTS

### Survey Response and Physician Profiling

The survey completion rate (all questions answered) was 7% in the United States and 16% in Europe (Table 1). A total of 629 physicians completed the survey, of 6428 approached, with similar characteristics between the global population, the United States, and Europe (Table 2). Overall, the proportion of cardiologists (57%) was higher than that of electrophysiologists (43%), with 80% reporting a specialization in AF. At the time of the survey, respondents had been qualified in their specialty for an average of 14.5 years. The most common clinical practice setting was a university hospital/clinic (40% global; 31% United States, 49% Europe) or a general community hospital/clinic (37% global; 33% United States, 40% Europe). Over a typical 3‐month period (before the COVID‐19 pandemic), the average total cardiology patient caseload, including all diagnoses and conditions, was 549; the average caseload of follow‐up patients was greater than the average caseload of new patients with AF (158 versus 94, respectively). Most respondents referred patients for ablation, rather than performing ablations themselves (55% versus 45%, respectively).

**Table 1:** Survey Response and Completion Rates

	United States			Europe		
	Total	Cardiologists	Interventional electrophysiologists	Total	Cardiologists	Interventional electrophysiologists
Invitations sent, n	4428	3382	1046	2000	1266	734
Responses,* n (%)	1075 (24)	721 (21)	354 (34)	716 (36)	423 (33)	293 (40)
Completed survey, n (%)	308 (7)	168 (5)	140 (13)	321 (16)	210 (17)	111 (15)

* Respondents who started the survey, including those who did not complete all questions.

**Table 2:** Physician Profiling and Demographics

Category	Global (N=629)	United States (n=308)	Europe (n=321)
Physician type, n (%)			
Cardiologist	360 (57)	168 (55)	192 (60)
Interventional electrophysiologist	269 (43)	140 (45)	129 (40)
Subspecialty, n (%)			
AF	501 (80)	231 (75)	270 (84)
Other	52 (8)	22 (7)	30 (9)
None	76 (12)	55 (18)	21 (7)
Length of time qualified in specialty			
3–25 y, n (%)	565 (90)	265 (93)	300 (86)
26–40 y, n (%)	64 (10)	43 (7)	21 (14)
Mean, y	14.5	15.0	14.0
Time spent on physician‐related activities, %			
Actively treating patients	89	93	86
Academia/research	6	4	8
Administration/other	5	3	7
Main clinical practice setting, n (%)			
General community hospital/clinic	230 (37)	102 (33)	128 (40)
University hospital/clinic	251 (40)	95 (31)	156 (49)
Primary outpatient practice/clinic	93 (15)	74 (24)	19 (6)
Private hospital/clinic	53 (8)	37 (12)	16 (5)
Other	2 (<1)	0	2 (1)
Typical patient caseload over 3 mo,* n			
Total cardiology patient caseload^†^	549	619	481
New patients with AF	94	82	105
Follow‐up patients with AF	158	175	141
Clinical activities, n (%)			
Prescribe drug treatments and perform ablation	284 (45)	150 (49)	134 (42)
Prescribe drug treatments and refer for ablation	345 (55)	158 (51)	187 (58)

Due to rounding, not all percentages add up to 100%.

AF indicates atrial fibrillation.

* Before the COVID‐19 pandemic.

^†^ Including all cardiology diagnoses and conditions.

### Physicians’ Attitudes Toward Guideline Use

Overall, 97% of respondents stated that they follow guidelines for their treatment decisions. Approximately half (49%) of the respondents primarily referred to the AHA/ACC/HRS guidelines^6^, ^7^ for their decision making, with 43% referring to the ESC guidelines.^5^ Overwhelmingly, US respondents chose the AHA/ACC/HRS guidelines^6^, ^7^ as their primary reference (96%), although 20% also referred to the ESC guidelines.^5^ European respondents generally chose the ESC guidelines^5^ as their primary reference (82%), with 32% also referring to the AHA/ACC/HRS guidelines.^6^, ^7^ Guidelines were chosen as the first and second most important nonpatient factor that influenced treatment strategy by 54% and 28% of respondents, respectively; other scientific literature was chosen by 23% and 41%, respectively. Most respondents (65%), including 58% of European respondents, were unsure whether the 2020 ESC guideline^5^ update had influenced their survey responses.

### AAD Choice in Specific Clinical Circumstances

AADs were chosen as a typical treatment choice across multiple patient comorbidity categories, with extensive use of amiodarone (selected by 60% to 80% of respondents across most queried comorbidities), despite guidelines recommending alternative first‐line agents in most settings. Sotalol use ranged from 18% to 46% between comorbidity categories, and dronedarone was used by 8% to 27% of respondents, while use of class Ic drugs was generally low. Estimated percentage of nonadherence for each agent was 93% for amiodarone, 61% for flecainide, 60% for sotalol, 48% for propafenone, 40% for dronedarone, and 25% for dofetilide.

#### No or Minimal Structural Heart Disease

**Figure 1:**
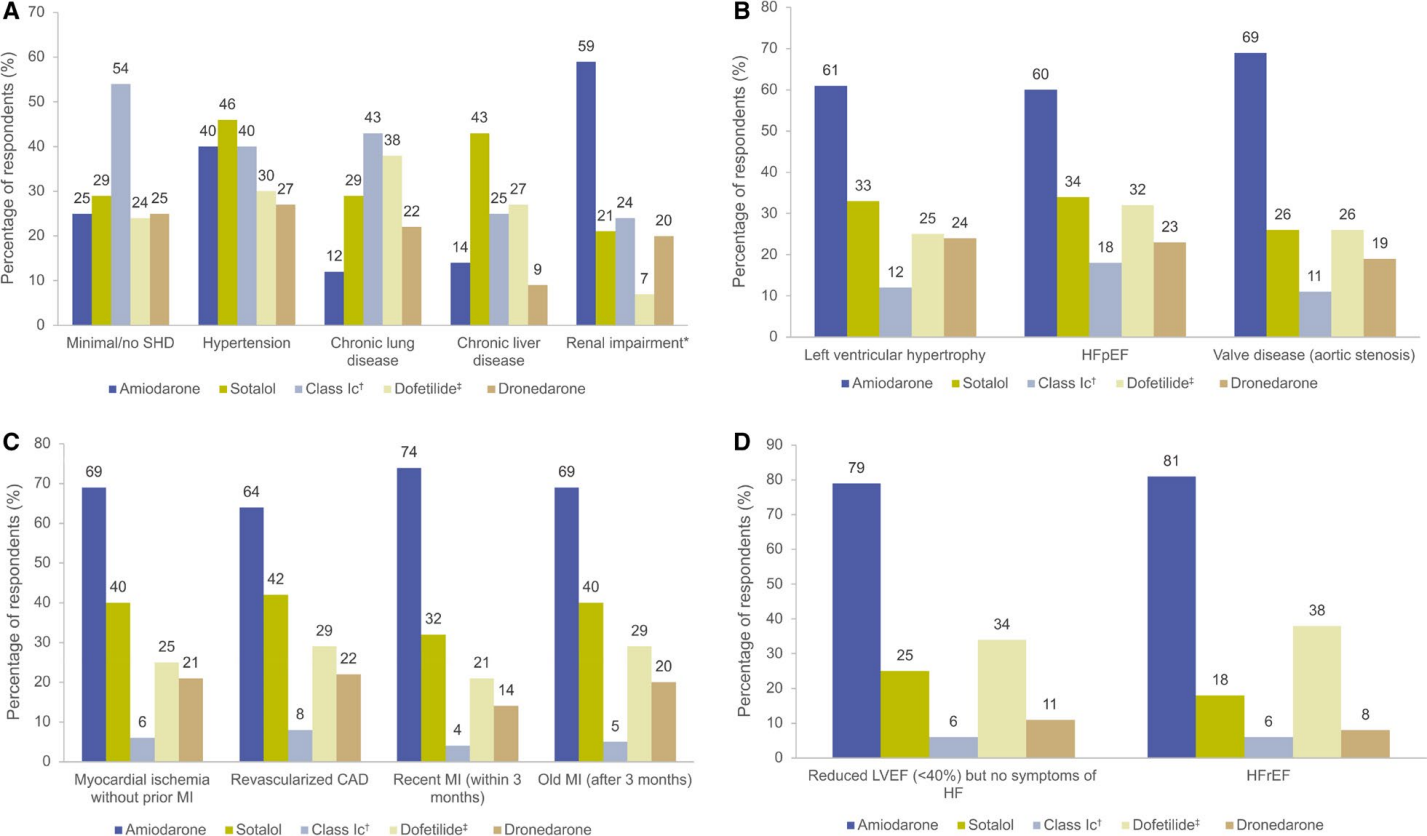
Proportion of respondents who selected AADs as a typical treatment choice in patients with specific comorbidities. **A**, Patients with minimal/no SHD and comorbidities unrelated to SHD. **B**, Patients with SHD and preserved ejection fraction. **C**, Patients with CAD. **D**, Patients with SHD and reduced ejection fraction. *Renal impairment defined as eGFR <60 mL/min per 1.73 m^2^; ^†^Average individual use of flecainide and propafenone; ^‡^US respondents only. AAD indicates antiarrhythmic drug; CAD, coronary artery disease; eGFR, estimated glomerular filtration rate; HF, heart failure; HFrEF, heart failure with reduced ejection fraction; HFpEF, heart failure with preserved ejection fraction; LVEF, left ventricular ejection fraction; MI, myocardial infarction; and SHD, structural heart disease.

In patients with no or minimal structural heart disease (SHD), 25% of respondents selected amiodarone as a typical treatment option (Figure 1A). Despite guidelines recommending that alternative agents should be considered first, class Ic agents were selected by the highest number of respondents (54%) in this patient group.

#### SHD With Preserved Ejection Fraction

**Figure 2:**
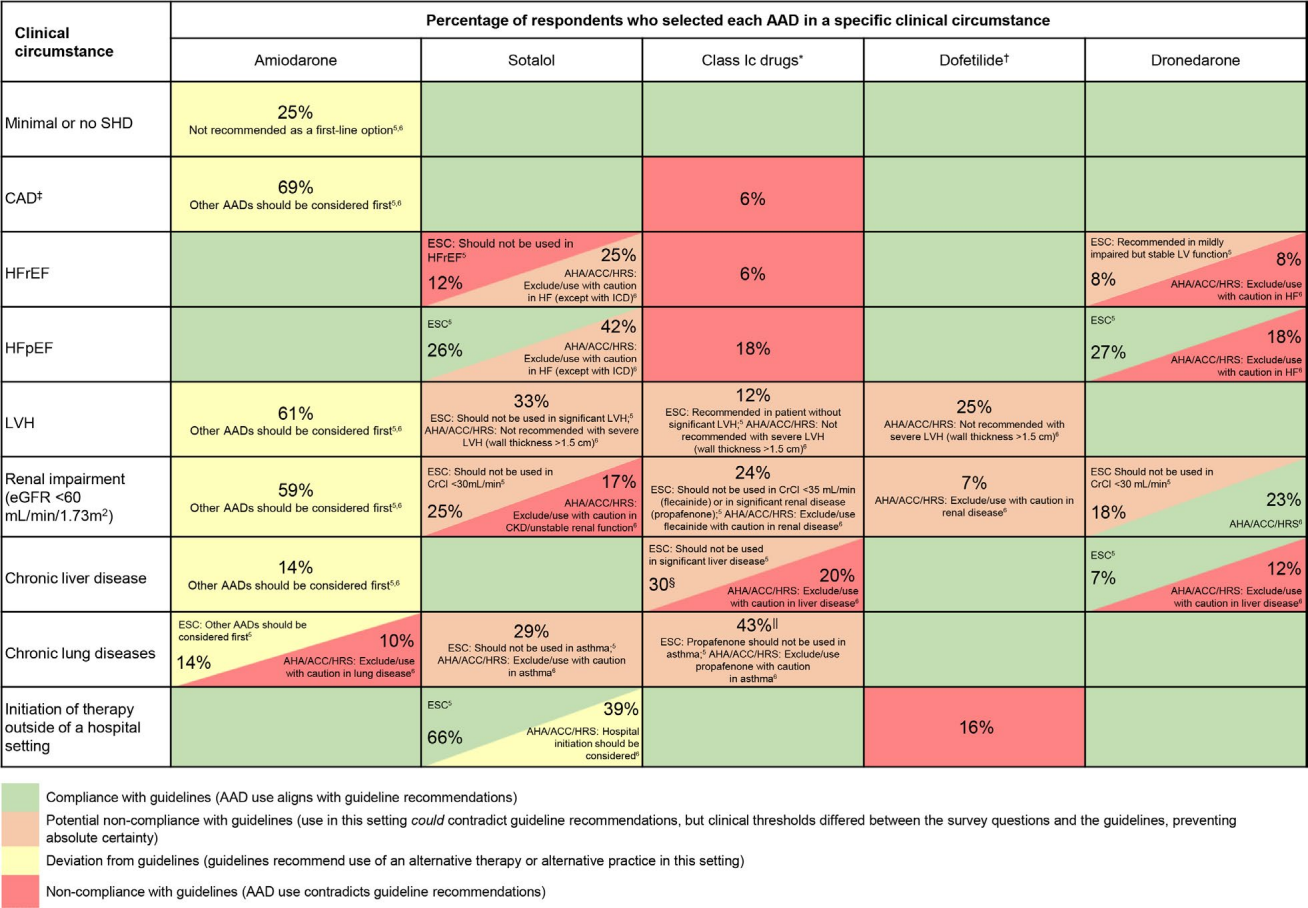
Degree of nonadherence to guidelines based on selection of AADs under specific clinical circumstances. Data shown describe the percentage of respondents who selected each AAD in specific clinical circumstances. The color codes describe the degree of adherence or nonadherence between survey responses and the 2014/2019 AHA/ACC/HRS^6^, ^7^ and 2020 ESC guideline recommendations.^5^ The text in the boxes corresponds to recommendations in the AHA/ACC/HRS^6^, ^7^ and ESC guidelines.^5^ Global data are shown for instances in which the AHA/ACC/HRS^6^, ^7^ and ESC^5^ guideline recommendations align. For instances in which AHA/ACC/HRS^6^, ^7^ and ESC^5^ guidelines have differing recommendations, both the US and European data are shown. *Average individual use of flecainide and propafenone (unless otherwise indicated); ^†^US respondents only (agent is not available in Europe and does not appear in ESC guidelines^5^); ^‡^Average use across myocardial ischemia, MI, and revascularized CAD; ^§^Flecainide 41%, propafenone 19%; ^‖^Data shown for propafenone only. AAD indicates antiarrhythmic drug; ACC, American College of Cardiology; AF, atrial fibrillation; AHA, American Heart Association; CAD, coronary artery disease; CKD, chronic kidney disease; CrCl, creatinine clearance; eGFR, estimated glomerular filtration rate; ESC, European Society of Cardiology; HF, heart failure; HFpEF, heart failure with preserved ejection fraction; HFrEF, heart failure with reduced ejection fraction; HRS, Heart Rhythm Society; ICD, implantable cardioverter defibrillator; LV, left ventricular; LVEF, left ventricular ejection fraction; LVH, left ventricular hypertrophy; MI, myocardial infarction; and SHD, structural heart disease.

Sotalol and class Ic agents were selected as a typical treatment choice in left ventricular hypertrophy (LVH) by 33% and 12%, respectively (Figure 1B). This could indicate potential noncompliance, as these agents are not recommended in patients with severe LVH (ESC guidelines^5^) or significant LVH (wall thickness >1.5 cm; AHA/ACC/HRS guidelines^6^, ^7^). In patients with HF with preserved ejection fraction, guidelines do not recommend use of class Ic agents, yet they were selected by 18% of respondents, indicating noncompliance (Figure 2).

#### Coronary Artery Disease

The guideline‐preferred AADs for use in patients with coronary artery disease (CAD) are dronedarone (IA) and sotalol (IIbA in ESC guidelines),^5^ plus dofetilide in the United States. However, class Ic agents were selected as a typical treatment choice in CAD (average across myocardial infarction presentations and revascularized CAD; Figure 1C) by 6% of respondents (numbers reported are the average use of flecainide and propafenone; Figure 2), indicating noncompliance with guidelines.

#### SHD With Reduced Ejection Fraction

Amiodarone is recommended for use in patients with HF with reduced ejection fraction (HFrEF) by both sets of guidelines and AHA/ACC/HRS recommendations^6^, ^7^ also include dofetilide in this setting. Dronedarone may be used in patients with mildly impaired but stable left ventricular function according to the ESC guidelines^5^ and was used by 9% of respondents. Notably, sotalol was selected by 18% of respondents, despite ESC guidelines^5^ not recommending it and AHA/ACC/HRS guidelines^6^, ^7^ advising to exclude or use with caution in this setting. Class Ic agents were used by 6% for patients with HFrEF (Figure 1D), which directly contradicts guidelines, indicating noncompliance (Figure 2).

#### Other Comorbidities

Class Ic drugs (43%) and sotalol (29%) were selected as typical choices in chronic lung disease (Figure 1A), which could indicate potential noncompliance, as guidelines recommend avoiding use in patients with asthma. In patients with renal impairment (estimated glomerular filtration rate <60 mL/min per 1.73 m^2^), sotalol was selected by 21% of respondents, which contradicts AHA/ACC/HRS guidelines^6^, ^7^ and may indicate potential noncompliance with ESC guidelines,^5^ which state that sotalol should not be used if creatinine clearance is <30 mL/min. Responses also suggested potential noncompliant use of class Ic drugs in chronic liver disease (25%) and renal impairment (24%) (Figure 2).

### Initiation of AAD Therapy

A notable number of respondents indicated they initiated sotalol therapy outside a hospital setting (53%). While this does not directly contradict recommendations, the 2014 AHA/ACC/HRS guidelines^6^ state that hospital initiation of sotalol should be considered. As such, these responses suggest deviation from guidelines.

A number of respondents (United States only) initiated dofetilide outside a hospital setting (16%). This does not follow the 2014 AHA/ACC/HRS guidelines^6^ or the US Food and Drug Administration labeling for this agent,^9^ which recommend inpatient initiation of dofetilide because of QT prolongation and risk of torsades de pointes.

### Monitoring of Patients Receiving AADs

**Figure 3:**
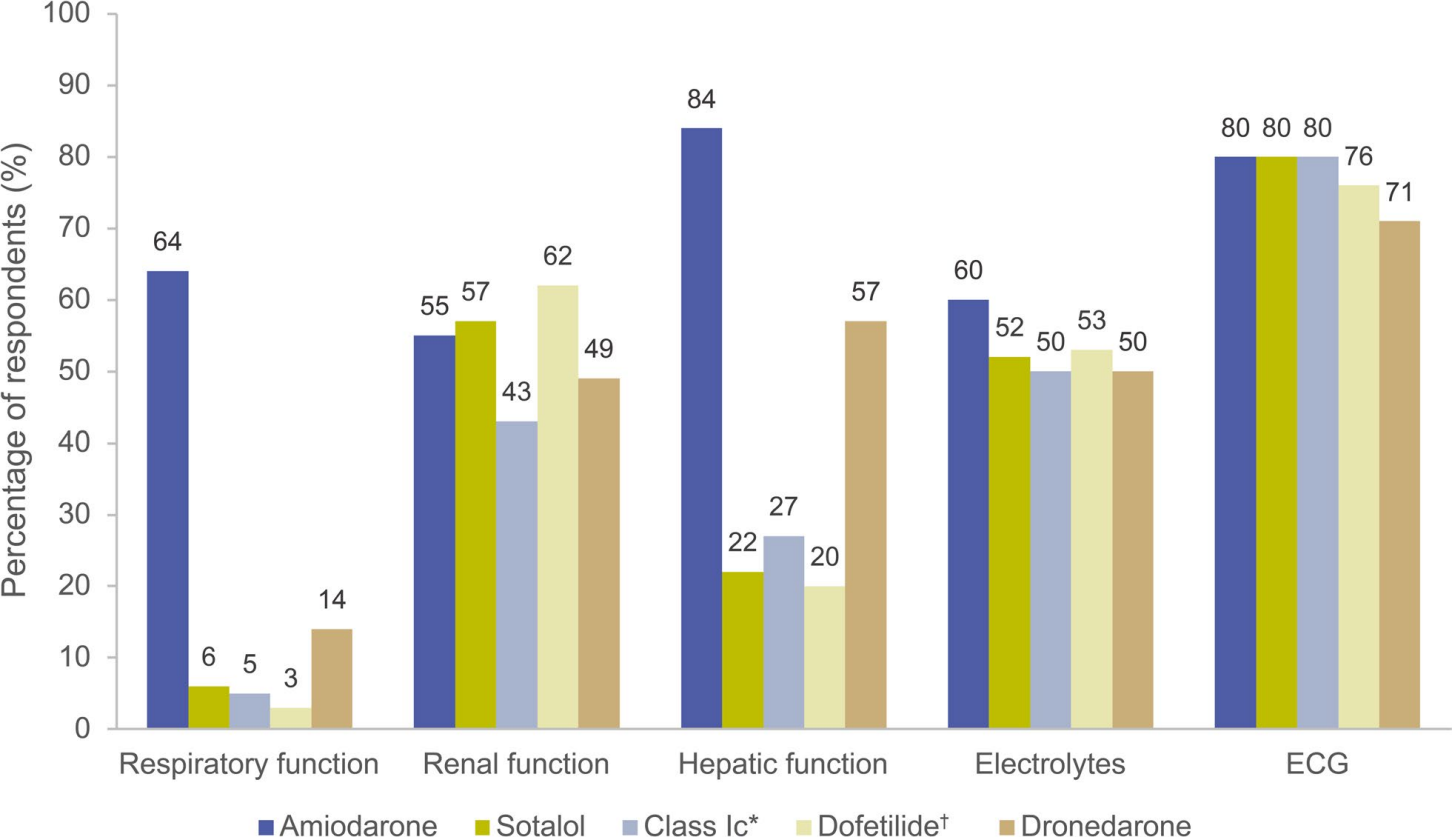
Proportion of respondents who routinely (at least annually) request investigations in patients with AF receiving AADs. Total respondent numbers varied slightly between drugs. *Average individual use of flecainide and propafenone; ^†^US respondents only. AAD indicates antiarrhythmic drug; and AF, atrial fibrillation.

Guidelines recommend close monitoring of proarrhythmic risk factors in individuals using AADs. In general, respondents requested routine investigations (at least annually) most often with amiodarone (Figure 3). ECGs were routinely requested by 80% of respondents with amiodarone, sotalol, and class Ic drugs. Routine requests for electrolyte monitoring was similar between all AADs, but notably low for sotalol (52%) and class Ic drugs (50%). Renal function monitoring was also notably low with sotalol (57%) and dofetilide (US respondents only; 62%), despite both guidelines recommending electrolyte and renal function monitoring for all patients receiving sotalol. Respiratory function monitoring was requested by 64% of respondents with amiodarone and 14% with dronedarone. Monitoring of hepatic function with amiodarone, dronedarone, and class Ic drugs was requested by 84%, 57%, and 27% of respondents, respectively.

### Use of Rhythm and Rate Control Strategies Across AF Subtypes

**Figure 4:**
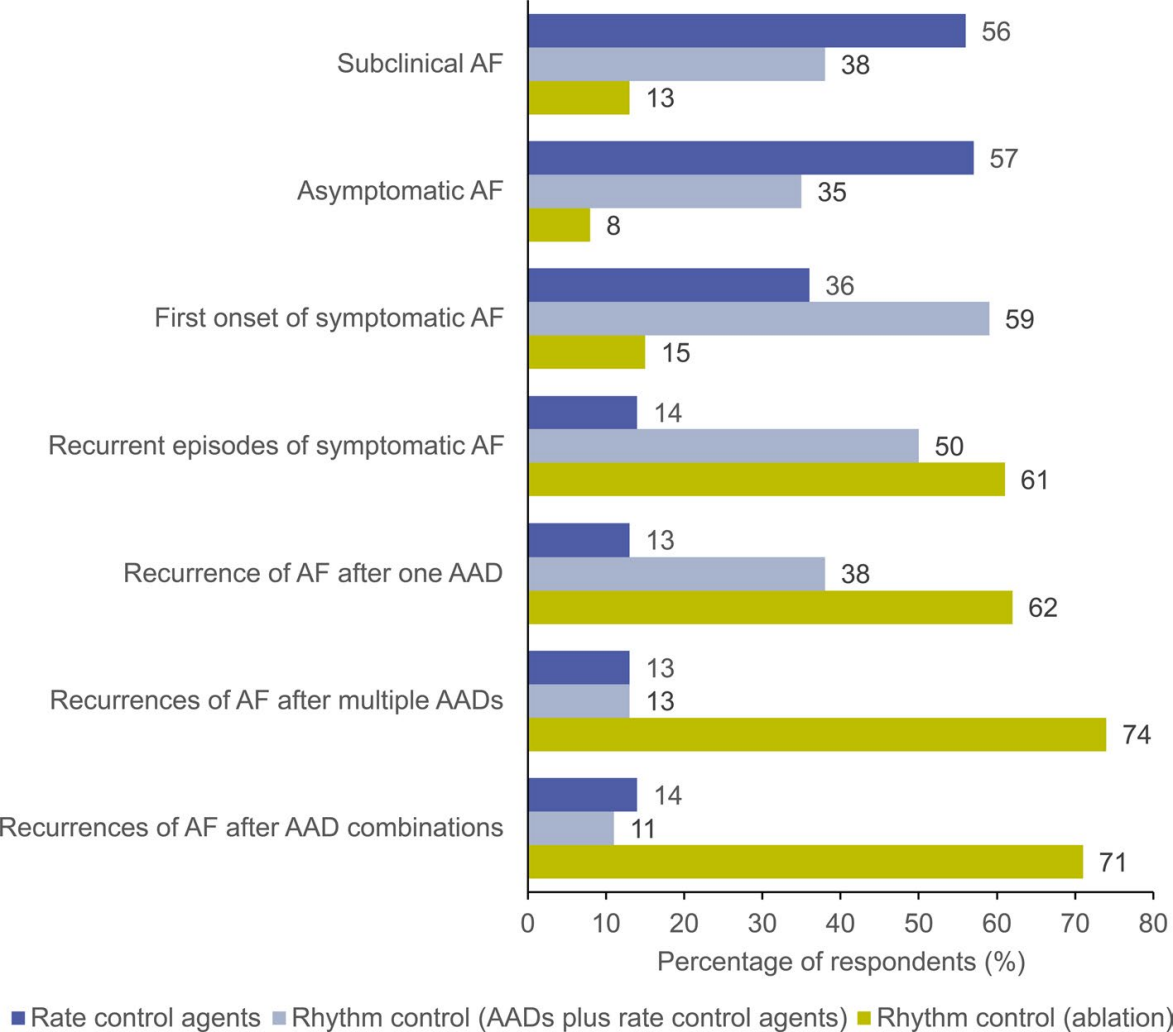
Proportion of respondents who selected rhythm and rate control strategies across AF subtypes. AAD indicates antiarrhythmic drug; and AF, atrial fibrillation.

Survey responses indicated notable variation in control strategy, dependent on AF subtype (Figure 4). Use of rate control agents was most frequent for asymptomatic and subclinical AF (57% and 56%, respectively). However, rhythm control strategies were also reported in these 2 AF groups, using AADs (35% and 38%, respectively) and performing ablation (8% and 13%, respectively). Ablation was the most common treatment strategy for recurrent episodes of symptomatic AF (61%), and its use increased with the prior failure of single (62%) or multiple AADs (74%) in preventing AF recurrence, as well as with prior failure of AAD combinations (71%) (Figure 4).

On average, AADs were primarily used more often as a first‐line strategy than rate control agents in paroxysmal AF (in 60% versus 32% of patients, respectively). For the management of persistent AF, rate control agents were primarily used over AADs (in 51% versus 42% of patients, respectively).

### Use of Drug Combinations

**Figure 5:**
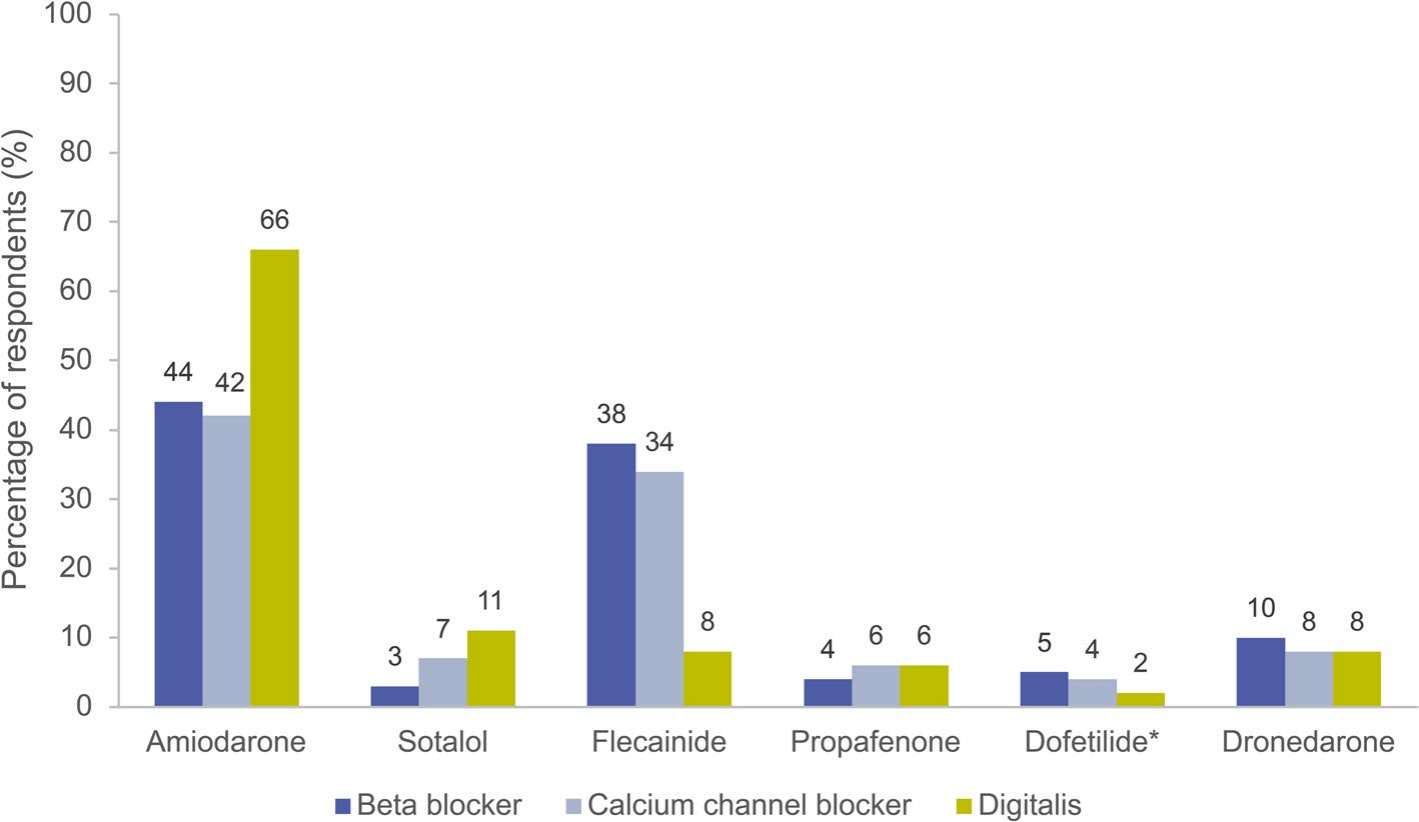
Proportion of respondents who selected different AADs in combination with rate control agents. Total respondent numbers varied slightly between each rate control agent. *US respondents only. AAD indicates antiarrhythmic drug.

Beta blockers (90%) were the most frequently used rate control agent in combination with an AAD for rhythm control, followed by calcium channel blockers (32%), and digitalis (19%). Drug combinations were most frequently used for the treatment of persistent AF (in 36% of patients), mixed persistent/paroxysmal AF (in 34% of patients) and paroxysmal AF (in 32% of patients). Amiodarone was the AAD most frequently selected in combination with a rate control agent, used by 66% of respondents in combination with digitalis, 44% with a beta blocker, and 42% with a calcium channel blocker (Figure 5).

The ESC guidelines^5^ recommend avoiding combinations of >1 AAD to minimize proarrhythmic risk. However, 10% reported that this was the most common type of combination regimen used for rhythm control. On average, respondents would try combinations of AADs (add‐on) in 20% of patients if they experienced a recurrence while receiving an AAD.

### Factors Influencing Therapy Selection

Despite guideline algorithms emphasizing safety first, efficacy was felt to be the most important nonpatient factor for selection of rhythm control therapy (48% ranked it first from a list of 9 general considerations; data not shown), while safety was considered the second most important factor (34%). Symptomatic status was ranked by 38% as the most important patient factor in guiding the choice of rhythm control therapy (data not shown). Overall, the combination of both antiarrhythmic properties and rate control properties in a single drug with multichannel effects, such as amiodarone, dronedarone, or sotalol, influenced 68% of respondents regarding their choice of AAD; 23% felt that their AAD choice was not influenced, and 9% were unsure.

### Regional Differences in Treatment Practices and Guideline Adherence

The largest difference in treatment practice overall between US and European respondents was the use of dofetilide in the United States (selected across patient subgroups, by 7% to 38% of respondents), whereas this agent is not marketed for use in Europe and was not selected by European respondents. This is likely to have led to regional disparities in the selection of other agents, most notably amiodarone, which was selected by significantly more European respondents (*P* < 0.05) across all SHD subgroups apart from in patients with LVH. However, sotalol was used more frequently by US respondents across most comorbidity categories, including LVH and HFrEF. Across all AADs used in both regions, routine investigations were generally requested by fewer US respondents than European respondents.

Regional differences were also seen in the degree of adherence to specific guideline recommendations (Table 3). Considering AAD usage clearly noncompliant with guidelines, class Ic agents were selected in HFrEF, and propafenone was selected in LVH by significantly more US than European respondents (*P* < 0.05). Additionally, significantly more European than US respondents selected flecainide in LVH (19% versus 11%, *P* < 0.05). Renal function monitoring with sotalol, which is recommended by guidelines, was performed by statistically significantly more US respondents than European respondents (64% versus 50%, respectively; *P* < 0.05), against the general trend for US practitioners to request fewer routine follow‐up investigations. Use of AADs for rhythm control was statistically significantly lower in US respondents than European respondents for asymptomatic AF (31% versus 39%, respectively; *P* < 0.05) and subclinical AF (33% versus 43%, respectively; *P* < 0.05), both deviating from guideline recommendations.

**Table 3:** Survey Responses Indicating Significant* Differences Between Proportions of US and European Respondents Reporting Specific Cases of Guideline Nonadherent Practice

Treatment practices, n (%)	United States (n=308)	Europe (n=321)
Treatments typically selected for patients with specific comorbidities		
HFrEF		
Class Ic^*^	24 (8)	12 (4)
Sotalol	77 (25)	38 (12)
CAD^‡^		
Amiodarone	186 (77)	248 (60)
LVH		
Class Ic^*^	36 (12)^†^	41 (13)^†^
Flecainide	35 (11)	60 (19)
Propafenone	36 (12)	22 (7)
Chronic liver disease		
Amiodarone	33 (11)	53 (17)
Dronedarone	37 (12)	21 (7)
Class Ic^*^	60 (20)	97 (30)
Renal impairment^‡^		
Class Ic^*^	62 (20)	88 (28)
Sotalol	52 (17)	80 (25)
Chronic lung disease		
Class Ic^*^	124 (40)	146 (46)
Sotalol	105 (34)	75 (23)
Routine investigations^§^ (at least annually)		
Electrolytes		
Flecainide	130 (44)	189 (60)
Propafenone	122 (41)	158 (53)
Hepatic function		
Dronedarone	134 (46)	206 (67)
Renal function		
Sotalol	191 (64)	152 (50)
Initiation of AADs outside of a hospital inpatient setting		
Sotalol	121 (39)	212 (66)
Use of AADs for rhythm control		
In asymptomatic AF	96 (31)	125 (39)
In subclinical AF	101 (33)	138 (43)

AAD indicates antiarrhythmic drug; AF, atrial fibrillation; CAD, coronary artery disease; HFrEF, heart failure with reduced ejection fraction; LVH, left ventricular hypertrophy; and MI, myocardial infarction.

* *P* < 0.05.

^†^ Average individual use of flecainide and propafenone.

^‡^ Average use across myocardial ischemia, MI and revascularized CAD.

^§^ Overall use of class Ic agents in this subgroup was similar across regions, but differences were seen in use of the individual agents.

^‖^ Defined as eGFR<60 ml/min/1.73 m^2^.

^#^ Total respondent numbers in US and Europe varied between all drugs.

## Discussion

The AIM‐AF physician survey extensively explored cardiologist and electrophysiologist treatment decisions regarding antiarrhythmic treatment for AF in 629 respondents. The response rate seen was in line with those previously reported from online physician surveys.^12^, ^13^, ^14^ The major finding from this study is that there is a high level of deviation, of varying degrees, from the 2020 ESC^5^ and 2014/2019 AHA/ACC/HRS guidelines^6^, ^7^; a particularly surprising result, since 97% of respondents stated that they follow guidelines and 54% felt that guidelines were the most important nonpatient factor influencing their treatment decisions. It is not possible to determine from these data whether this degree of deviation results from an unexpectedly high level of adaptation of treatment to suit individual patients or reflects a serious knowledge gap among treating physicians. However, the survey questions were worded to ascertain general treatment practices, so respondents would not be expected to select answers based on individual patient circumstances.

While deviations from guidelines may be reasonable in select clinical circumstances, a high degree of nonadherence raises concerns regarding patient safety.^15^, ^16^ Despite the growing use of ablation, appropriate AAD use is an increasingly important issue in clinical practice, as one study found that antiarrhythmic prescriptions nearly tripled between 2004 and 2016 in the United States, with the most substantial increases observed for amiodarone, sotalol, flecainide, and dofetilide.^17^ As the clinical presentations of AF evolve over time, and guidelines are regularly updated in line with new evidence, important safety questions arise over the extent to which physicians are keeping abreast of such updates, particularly with regard to AAD use.

The choice between rate control and rhythm control in the treatment of patients with AF was widely debated until the publication of the AFFIRM (Atrial Fibrillation Follow‐up Investigation of Rhythm Management) study, which found no clear survival advantage when using a rhythm control strategy compared with rate control.^18^ However, more recent data have shown greater improvement in quality of life,^19^ functional status,^19^, ^20^, ^21^, ^22^ exercise tolerance,^23^ and also reductions in both symptoms^24^ and symptomatic HF incidence^25^ with restoration of sinus rhythm (using ablation or AADs) compared with rate‐controlled AF. Moreover, The Early Treatment of Atrial Fibrillation for Stroke Prevention Trial found that early comprehensive rhythm control reduced the risk of adverse cardiovascular outcomes (a composite of death from cardiovascular causes, stroke, or hospitalization with worsening of HF or acute coronary syndrome) versus usual care, demonstrating that AADs remain an important treatment option for many patients with AF.^26^

A key finding in this survey was the factors that drive contemporary AAD selection. Both the ESC^5^ and AHA/ACC/HRS guidelines^6^, ^7^ advocate a safety‐based algorithm for AAD selection; however, almost half (48%) of respondents in our survey considered efficacy to be the most important consideration for selection of rhythm control. This finding has implications for patient management and likely explains the high use of amiodarone regardless of the clinical scenario.

Despite the well‐known organ toxicity and complex drug interaction profile associated with its use, amiodarone was frequently chosen as a typical treatment across multiple patient comorbidity categories, although both guidelines recommend consideration of other AADs first. However, routine monitoring via all queried parameters was considerably higher with amiodarone than other AADs, suggesting that respondents were aware of the increased safety considerations related to amiodarone. While class Ic drugs were mainly used in patients with minimal or no SHD, a notable proportion were also used in patients with CAD, HFrEF/HF with preserved ejection fraction, or LVH, which is contrary to guidelines and increases the risk of potentially life‐threatening proarrhythmia. In the ORBIT‐AF (Outcomes Registry for Better Informed Treatment of Atrial Fibrillation), 44% of investigators used a class Ic agent in patients with CAD, representing documented noncompliance with guidelines. Additionally, 35% used amiodarone as a first‐line therapy in patients without HF or LVH, representing the second most common instance of noncompliance in the ORBIT‐AF registry.^27^

In our study, the extent to which sotalol was selected as a typical treatment in patients with LVH (33%), renal impairment (21%), and HFrEF (18%) was of concern. Similar results were seen in the GWTG‐AFib (Get With The Guidelines—Atrial Fibrillation) study, where 20% and 17% of patients, respectively, received sotalol in the presence of HF and LVH.^28^ In the 2020 update to the ESC guidelines,^5^ sotalol was downgraded from a class I to a class IIb recommendation on the basis of evidence of increased mortality compared with placebo^29^ and other AADs.^30^, ^31^ There was no downgrading of sotalol in the 2019 update to the AHA/ACC/HRS guidelines,^7^ perhaps a contributing factor to greater overall use of sotalol in the United States than in Europe, but close monitoring in line with the ESC guidelines is advised.^5^ The 2020 update to the ESC guidelines include no specific recommendations with regard to sotalol initiation in hospital^5^; however, according to the AHA/ACC/HRS guidelines, hospital initiation of sotalol should be considered, although it is acknowledged that there is considerable experience of sotalol initiation in patients with a low risk of torsades de pointes outside of a hospital.^6^, ^7^ It is perhaps unsurprising, therefore, that our study found that statistically significantly fewer US respondents initiated sotalol outside of a hospital compared with European respondents (39% versus 66%; *P* < 0.05). With dofetilide, however, despite the clear guideline recommendation for hospital initiation, 16% of US respondents still initiated dofetilide outside of hospital. Although a recent study found that safe outpatient initiation of dofetilide was possible with intensive monitoring,^32^ this involved a very small patient cohort and should not yet inform clinical practice.

Our survey results revealed that a rhythm control strategy is being frequently used to treat asymptomatic and subclinical AF, with only 38% of respondents ranking symptomatic status as the top factor for influencing selection of rhythm control rather than rate control. While the use of early rhythm control is gaining more interest and supporting evidence,^26^ the ESC and AHA/ACC/HRS guidelines recommend that rhythm control should be confined only to otherwise symptomatic patients.^5^, ^6^, ^7^ Of note, the Euro Heart Survey also found that rhythm control strategies were used in 44% to 46% of asymptomatic patients.^33^

This study extensively explored physicians’ attitudes toward antiarrhythmic therapies and their treatment practices in patients with AF. Strengths of the AIM‐AF study include the fact that responses were gathered from cardiology physicians across several countries, the majority of whom considered AF to be their subspecialty. Additionally, the survey explored physicians’ attitudes to therapy selection, which provided a better understanding of physicians’ decision‐making processes.

A key limitation of the study is that data were dependent on the accurate reporting of information by the respondents, which may have been subject to recall bias. Additionally, the survey sample was taken from physicians who were part of the M3 Global International Market Research Panel, and only from 4 European countries. The survey completion rate was rather low (7% in the United States and 16% in Europe), as is often the case with wide‐reaching surveys such as the one used in this study; as such, the respondents may not be wholly representative of the general population of physicians treating AF. However, it is likely that physicians who did respond to the survey were more representative of high‐quality standards of care, which is particularly noteworthy in this context, given that adherence to guidelines was unsatisfactory. Another study limitation lies in the fact that this survey did not consider AAD dosing, which can condition both safety and efficacy of therapy and, as such, could have influenced physician responses. Furthermore, the threshold values assigned for certain questions were different than thresholds cited in the guidelines; for instance, guidelines recommend against using sotalol in patients with creatinine clearance <30 mL/min, while the survey classified renal impairment as estimated glomerular filtration rate <60 mL/min per 1.73 m^2^. Results were not stratified by degree of renal impairment; therefore, it remains difficult to accurately estimate the number of respondents who are noncompliant in this regard. Both the lack of information on dosing and the inclusion of data on potential noncompliant prescribing could have had the effect of overinflating the nonadherence rates calculated for each agent, as each could result in compliant practice being scored as nonadherent. Furthermore, these rates do not include any weighting for the degree of deviation from the guidelines or the potential outcomes of nonadherence; for example, the use of a contraindicated agent in a patient with HF, which could severely compromise patient safety, has the same weight as a guideline‐compliant dose reduction in a patient with renal impairment.

## Conclusions

Across the United States and Europe, many physicians considered guidelines to be the most important nonpatient factor influencing treatment decisions with regard to AAD use. However, nonadherence with guideline recommendations was common, and responses indicated notable noncompliance and potential noncompliance, which may compromise patient safety. Further research to better understand physicians’ reasons for nonadherence and interventional opportunities to improve adherence to guidelines is warranted.

## Sources of Funding

The AIM‐AF study and medical writing and editorial support for the development of this manuscript was funded by Sanofi. The funder had no role in either the study design, data collection, data analysis, data interpretation, or the decision to publish the study.

## Disclosures

Dr Camm received personal fees from AltaThera, Sanofi, Abbott, Boston Scientific, Medtronic, and Menarini. Dr Blomström‐Lundqvist received honoraria from Bayer, Boston Scientific, Correvio, Medtronic, Milestone, MSD (Merck & Co.), Sanofi, Pfizer, and Phillips. Dr Boriani received speaker fees from Bayer, Boehringer Ingelheim, Boston, and Medtronic. Dr Goette received speaker fees from Abbott, AstraZeneca, Berlin Chemie, Bayer, Bristol‐Myers Squibb‐Pfizer, Boehringer Ingelheim, Daiichi‐Sankyo, Medtronic, Novartis, Omeicos, and Sanofi; and funding from the European Union Horizon 2020 (Grant No. 965286). Dr Kowey is a consultant for Sanofi. Dr Merino received personal fees from Boston Scientific, Microport, and Sanofi. Dr Piccini received grants for clinical research from Abbott, AHA (American Heart Association), Association for the Advancement of Medical Instrumentation, Bayer, Boston Scientific, and Philips; and consultant fees from Abbott, AbbVie, Ablacon, AltaThera, ARCA Biopharma, BIOTRONIK, Boston Scientific, LivaNova, Medtronic, Milestone, MyoKardia, ElectroPhysiology Frontiers, Pfizer, Respicardia, Sanofi, Philips, and UpToDate. Dr Saksena is an advisory board/research panel member for Sanofi and received research grants from Abbott and Sanofi. Dr Reiffel is an investigator for Janssen, Medtronic, and Sanofi; and a consultant for Acesion, Amarin, Correvio, Medtronic, and Sanofi.

## Supporting information

Table S1
