# Supplementary material for: AIM‐AF: A Physician Survey in the United States and Europe
Source: J Am Heart Assoc. 2022 Mar 4;11(6):e023838. doi: 10.1161/JAHA.121.023838 (PMC9075271; doi:10.1161/JAHA.121.023838)
Supplement: Supplementary file 1 — Table S1 [file JAH3-11-e023838-s001.pdf]

SUPPLEMENTAL MATERIAL

Table S1. List of survey questions in the AIM-AF study

Question number	Question	Responses
**Screening/physician demographics**		
S1	Firstly, in which country is your practice located?<br>*Please select one*	1. US<br>2. UK<br>3. Germany<br>4. Italy<br>5. Sweden<br>Other
S2a	What is your current primary medical interest?<br>*Please select all that apply*	1. Clinical cardiologist (non-interventional)<br>2. Non-interventional cardiac electrophysiologist<br>3. Interventional cardiac electrophysiologist<br>Other
S2b	Do you have any sub-specialty or areas of special interest?<br>*Please select one*	1. Atrial fibrillation (AF)<br>2. Other, please specify<br>None
S3	How many years have you been qualified in your specialty?<br>*Please indicate to the nearest year*	______years
S4	Approximately what percentage of your time is spent in the following activities?<br>*Please type % for each row*	______% actively treating patients<br>______% academic / research<br>______% admin / other
S5	In a typical 3 months (i.e. prior to the COVID-19 pandemic), how many patients with AF do you see?<br>*Please specify both new and existing AF patients*	______new patients<br>______existing/ongoing patients

S7a&b	In a typical 3-month period, on how many patients with AF do you conduct / refer an ablation procedure? Roughly what proportion of your total caseload of AF patients does this represent?<br>*Please type in number*	_____ per month<br>_____% of my AF patients
S8	Which of the following best describes your role in the treatment of patients with AF?<br>*Please select one only*	1. I prescribe drug treatments and ablate<br>2. I prescribe drug treatments and refer for ablation<br>3. I do not prescribe drug treatments nor perform ablation
S9	Do you agree with these terms and conditions?<br>*Please select one*	1. I agree<br>2. I do not agree
S10	***Adverse event reporting***<br>This study is funded by a pharmaceutical company and for this reason we are required to pass on any possible Adverse Events, Product Complaints and Special Reporting Situations. The details of these will be reported anonymously unless you agree to disclose your personal details, only and exclusively for the purpose of follow-up by the client's drug safety team. Please select one of the options below:<br>*Please select one*	1. I would like to proceed and agree to be contacted by the drug safety team for follow-up<br>2. I would like to proceed but do not wish to be contacted by the drug safety team for follow-up<br>3. I do not wish to proceed
S11	Please select which region/area you work in.<br>*Please select one*	Options were provided in an appendix
**Section A: Setting and caseload**		
A1	A1a Which health care settings do you spend your time at?<br>*Please tick all that apply*<br><br>A1b Please indicate your main practice setting.	1. General community hospital/clinic (i.e. public or government hospital)<br>2. University hospital/clinic<br>3. Primary outpatient practice/clinic<br>4. Private hospital/clinic<br>5. Other (please specify)
A2a	For your main practice setting, approximately how many practitioners (including yourself) are there in your department?	1. Clinical cardiologists<br>2. Non-invasive cardiac electrophysiologist<br>3. Cardiac invasive electrophysiologists<br>4. Internists

	Type in number for each row	5. Fellows<br>6. Clinical pharmacologists<br>7. Physician assistants/nurse practitioners
A2b	How are physician assistants/nurse practitioners primarily involved in the treatment of AF patients in your practice?<br><br>*Select all that apply*	1. Initiation of rate control treatments<br>2. Initiation of antiarrhythmic drugs (AADs)<br>3. Repeat prescriptions<br>4. Ongoing follow-up of patients<br>5. No role
A3a<br>A3b<br>A3c	Thinking about the patients you would see in a typical three-month period (i.e. prior to the COVID-19 pandemic):<br><br>What is your typical total cardiology patient caseload? *This should be overall and include all diagnoses and conditions*<br><br>What is your typical caseload of new patients with AF?<br><br>And what is your typical caseload of follow-up patients with AF?<br><br>*Please type number below:*	In a typical three-month period…<br><br>_____ total cardiology patient caseload<br>_____ new patients with AF<br>_____follow-up patients with AF
A4	Thinking about your AF patient caseload ([pipe number from A3b&c "AF patients"] patients), what percentage fall into each of the following subgroups?<br><br>*Please type % for each row*	1. First onset AF: AF presenting for the first time and not yet classified as paroxysmal, persistent, or permanent<br>2. Paroxysmal AF: Self-terminating, in most cases within 48 hours. Some AF paroxysms may continue for up to 7 days. AF episodes that are cardioverted within 7 days should be considered paroxysmal<br>3. Persistent AF: AF that lasts longer than 7 days, including episodes that are terminated by cardioversion, either with drugs or by direct current cardioversion, after 7 days or more<br>4. Mixed paroxysmal and persistent

		5. Long-standing persistent AF: Where the patient has had continuous AF for a year or longer, but rhythm control will be tried<br>6. Permanent AF: Where AF is present continuously for more than one year but no rhythm control will be attempted
A5	Thinking about your AF patient caseload [pipe number from A3b&c "AF patients"] patients, what percentage would you define as subclinical AF detected on an implantable device (pacemaker, implantable cardioverter defibrillator, loop recorder) or a wearable device (watch, phone, etc.)?<br>*Please type % for each row*	1. Implantable device-detected subclinical AF ____%<br>2. Wearable device-detected subclinical AF ____%
A6	Of your AF patient caseload [pipe number from A3b&c "AF patients"] patients, approximately what percentage fits into the following categories when you first see them?<br>*Please type % for each row*	1. Inpatient __%<br>2. Day case (in hospital)__%<br>3. Outpatient (clinic) __%
**Section B: Treatment journey**		
**Information**	**Questions designed to identify the typical treatment approaches of physicians for their patients with AF, with a focus on the use of oral antiarrhythmic drugs (AADs) and what influences their decision making.**	
B1	In what percentage of your patients with AF do you opt for each main strategy as first-line (after dealing with anticoagulation)?<br><br>*Please type % for each row*	COLUMNS:<br>1. Paroxysmal AF<br>2. Persistent AF<br><br>ROWS:<br>1. Primarily heart rate control only<br>2. Primarily rhythm control (with drugs)<br>3. Other, please specify
B2	What factors influence/guide your choice of rhythm control rather than rate control?<br>*Please rank all options within each category*	<u>Non-patient factors:</u><br>1. Guidelines<br>2. Previous personal experience<br>3. Scientific literature<br>4. Advice from colleagues<br>5. Other, please specify

		<u>Patient factors:</u><br>1. Age of patient<br>2. Early onset of AF<br>3. Symptomatic status<br>4. Paroxysmal rather than persistent AF<br>5. Absence of structural heart disease<br>6. Presence of heart failure<br>7. Co-morbidities<br>8. Compliance<br>9. Patient preference<br>10. Other, please specify ________
B3	For what types of AF do you prefer to use (oral) antiarrhythmic drugs (AADs) as first line rather than ablation therapy?<br>*Please select all that apply*	1. Asymptomatic recurrent AF<br>2. Mildly symptomatic but infrequent paroxysmal AF<br>3. Highly symptomatic infrequent paroxysmal AF patient<br>4. Frequent symptomatic paroxysmal AF<br>5. Infrequent symptomatic persistent AF<br>6. Frequent symptomatic persistent AF (2 or more cardioversions in the past year)<br>7. Long-standing symptomatic persistent AF (a year or longer)<br>8. Other, please specify ____<br>9. No types of AF in particular
B4	How would you typically treat patients with subclinical (asymptomatic, detected by chance) AF, if at all?<br>*Please select one answer*	1. Primarily rate control<br>2. Primarily rhythm control (with drugs)<br>3. No rate or rhythm treatment
B5	What factors influence your preference for (oral) AADs rather than the alternative of ablation therapy?<br>*Please rank the top 5 influences.*<br><br>*Click or drag to place your top 5 in rank order, where 1=most influential*<br>*If any items do not influence you, do not rank them*	1. Presence of heart failure (HFrEF)<br>2. Other severe comorbidities<br>3. Potential for procedure-related complications<br>4. Old age of the patient<br>5. Patient preference<br>6. Cost/reimbursement<br>7. Concerns about ablation efficacy in general (dilated left atrium, time in persistent AF)<br>8. Long AF duration<br>9. ESC and ACC/AHA/HRS algorithms emphasize safety first over efficacy

		10. Need for medication for other conditions (patient is taking medication anyway)<br>11. Comorbidities that shorten survival<br>12. Other, please specify_____
B6	When choosing a particular (oral) AAD, please rank the top 5 considerations that broadly influence your choice of AAD.<br><br>*Click or drag to place your top 5 in rank order, where 1=most important*<br>*If any items do not influence you, do not rank them*	1. Efficacy<br>2. Safety<br>3. No need for hospitalization at initiation<br>4. Comfort with the drug based on prior experience<br>5. Drug–drug interaction<br>6. Cost/reimbursement<br>7. Patient comorbidities<br>8. Patient preference<br>9. Need for ongoing electrocardiogram or laboratory monitoring<br>10. Other, please specify ________
B7	When prescribing an AAD, does the regulatory agency approval of a drug for a specific rhythm control indication influence your decision regarding the use of that drug?<br>*Please select one*	1. Yes<br>2. No<br>3. Not sure
B8	Thinking in more detail about efficacy and safety considerations when prescribing an AAD, please rank the top 5 considerations that influence your choice of AAD.<br><br>*Click or drag to place your top 5 in rank order, where 1=most important*<br>*If any items do not influence you, do not rank them*	1. Efficacy in reducing mortality and CV hospitalizations<br>2. Efficacy in terms of % of sinus rhythm maintenance at long term after electrical CV event<br>3. Low risk of atrial proarrhythmia (e.g. 1:1 atrial flutter)<br>4. Low risk of ventricular proarrhythmia<br>5. Low risk of major cardiovascular adverse effects<br>6. Low risk of major non-cardiovascular adverse effects (pulmonary, hepatic, thyroid, neurologic)<br>7. Other, please specify ________
B9	Does the combination of both antiarrhythmic and rate control properties in a single drug influence your choice of AAD?	1. Yes<br>2. No<br>3. Not sure
B10	When do you consider an AAD as not working?<br><br>*Please select all that apply*	1. Single recurrence<br>2. Multiple recurrences of symptomatic episodes<br>3. Need for hospitalizations<br>4. High daily burden

		5. Other, specify ______
B11	In some cases, ablation may take place first-line prior to prescribing any AAD (Class I and III AADs). Why is this?<br>*Please select all that apply*	1. Prefer to conduct ablation as early as possible to prevent progression of AF<br>2. I would never conduct ablation first line *(exclusive)*<br>3. Concerns about AAD efficacy/belief of higher efficacy with ablation<br>4. Concerns about AAD safety<br>5. Best treatment for paroxysmal AF<br>6. Avoiding anticoagulation<br>7. Special conditions: Comorbidities e.g. heart failure<br>8. Special conditions: Sinus node dysfunction<br>9. Special conditions: Age of the patient<br>10. Drug–drug interaction<br>11. Special conditions: Exercise/athletic considerations<br>12. Avoidance of long-term drug therapy<br>13. Patient preference<br>14. Cost/reimbursement/beneficial economic profile for practice or hospital<br>15. Other, please specify____
B12	Of the answers you selected, please pick the top 3 reasons for why ablation may take place first-line prior to prescribing any AAD (Class I and III AADs)?<br><br>*Click or drag to place your top 3 in rank order, where 1=most important*<br>*If any items do not influence you, do not rank them*	1. [Answers piped from B11]
B13	Does your center focus on ablation or AADs as a first-line treatment recommendation, or are both drugs and ablation options used as first-line?<br>*Select one*	1. Focus on ablation first-line<br>2. Focus on AAD as first-line<br>3. Offer both drugs and ablation first-line
B14a<br><br>B14b	Thinking about the following circumstances/comorbidities that AF patients often present with…	COLUMNS (DRUG SHORT LIST):<br>1. Amiodarone<br>2. Dronedarone<br>3. Flecainide<br>4. Propafenone

	Which AAD(s) would you typically use in these patients?<br>*Select all per row*<br><br>And of the ones you use, which do you use most of all?<br>*Please select which of the AAD(s) you are most likely to prescribe for each comorbidity*	5. Sotalol<br>6. Dofetilide (US only)<br>7. Other AAD, please specify<br><br>ROWS: AF patients with…<br>1. Minimal or no structural heart disease<br>2. Heart failure with reduced left ventricular function (with LVEF <40%)<br>3. Heart failure with preserved left ventricular function<br>4. Reduced left ventricular function (LVEF <40%) but no symptoms of heart failure<br>5. Left ventricular hypertrophy<br>6. Hypertension<br>7. Valve disease i.e. aortic stenosis<br>8. Myocardial ischemia without prior myocardial infarction<br>9. Revascularized coronary artery disease patient<br>10. Recent myocardial infarction (within 3 months)<br>11. Old myocardial infarction (after 3 months)<br>12. Renal impairment (eGFR <60mL/min/1.73m^2)<br>13. Chronic lung diseases<br>14. Chronic liver disease
B15	How would you manage most patients in the following categories?<br><br>*Please select all that apply in each row (for each patient type)*	ROWS:<br>1. Implantable-device-detected or subclinical AF<br>2. Asymptomatic AF<br>3. First attack of symptomatic AF<br>4. Recurrent episodes of symptomatic AF<br>5. Recurrence after one AAD<br>6. Recurrences after multiple AADs<br>7. Recurrences after AAD combinations<br><br>COLUMNS:<br>1. Drug rate control alone (no rhythm control)<br>2. Ablation for rate control (AV node ablation) and pacemaker implantation<br>3. Drug rhythm control (plus rate control with drugs)

		4. Ablation for rhythm control
B16a<br><br>B16b	Which of the following guidelines do you follow for the treatment of patients with AF?<br>*Select all that apply*<br><br>Which is the MAIN one that you follow / that is most important for your decision making?<br>*Select one*	1. American College of Cardiology (ACC)/American Heart Association (AHA)/Heart Rhythm Society (HRS)<br>2. Canadian Cardiovascular Society (CCS)<br>3. European Society of Cardiology (ESC)<br>4. National Institute for Health and Care Excellence (NICE) guidelines<br>5. Other national/local guidelines, please specify _______<br>6. Hospital guidance/protocol<br>7. I do not follow any particular treatment guidelines
B17	Of the following AAD drugs, which method of initiation do you use in most of your patients?<br><br>1. Typically initiate in hospital<br>2. Outpatient initiation with intensive ECG monitoring<br>3. Initiate out of hospital with only a routine clinic appointment after initiation<br><br>*Select one option per drug*	ROWS:<br>1. Amiodarone<br>2. Dronedarone<br>3. Flecainide<br>4. Propafenone<br>5. Sotalol<br>6. Dofetilide (US only)<br><br>COLUMNS:<br>1. Typically initiate in hospital<br>2. Outpatient initiation with intensive ECG monitoring<br>3. Initiate out of hospital with only a routine clinic appointment after initiation
B18	In what proportion of patients do you use these methods to monitor for recurrences?<br>*Please type % for each (several methods may be used, i.e. does not need to add up to 100%)*	1. Symptoms<br>2. Patient self-check of pulse<br>3. 12-lead ECGs in the clinic<br>4. Ambulatory Holter recordings or patch ECG recordings<br>5. Loop recorders<br>6. Watch plethysmographs<br>7. Watch ECGs<br>8. Smart phone ECGs<br>9. Event recorders (Zio, Bardy, etc)<br>10. Other, please specify _______<br>11. No routine monitoring for recurrence

B19	How often do you use an implantable loop recorder for monitoring in each of the following situations?<br>*Please select one per row*	ROWS:<br>1. Documentation of AF burden pre-ablation<br>2. Assessment of AT/AF occurrence/recurrence post-ablation<br>3. Symptom diagnosis<br>4. Assessment of AAD efficacy<br>5. Evaluation of rate control<br><br>COLUMNS:<br>1. Always (76–100% of patients)<br>2. Often (51–75%)<br>3. Sometimes (26–50%)<br>4. Rarely (1–25%)<br>5. Never (0%)
B20	How do you routinely verify a) heart failure and b) ischemic heart disease before undertaking AF AAD treatment?<br><br>*Select all that apply*	COLUMNS:<br>a) Heart failure<br>b) Ischemic heart disease<br><br>ROWS:<br>1. Functional stress testing<br>2. Echocardiography e.g. for assessment of LA size and LVEF, etc.,<br>3. Other imaging (cardiac CT, MRI, coronary angiography)<br>4. Other, please specify ________<br>5. Do not routinely verify [exclusive]
**Section C: Prescribing/treatment practices**		
**Information**	**Questions designed to focus in more detail on specific treatment practices.**	
C1	Please indicate the % of your patients with AF who would receive each treatment approach as first-line treatment.<br><br>*Please type % of patients for each column*<br>*Keep thinking about your [A3b+c] patients as your total AF population*	COLUMNS (pipe in numbers in each subgroup from A4/A5):<br>1. First onset AF (unclassified)<br>2. Paroxysmal AF<br>3. Persistent AF<br>4. Mixed paroxysmal and persistent<br>5. Long-standing persistent AF<br>6. Permanent AF<br>7. Device/wearable-detected asymptomatic AF

		ROWS:<br>1. Drug rate control alone (no rhythm control)<br>2. Ablation for rate control (AV node ablation) and pacemaker implantation<br>3. Drug rhythm control (plus rate control with drugs)<br>4. Ablation for rhythm control<br>5. Other<br>6. None of the above
C2a	How often do you use beta-blockers for a) rate control and b) rhythm control?<br>*Please select one per row*	COLUMNS:<br>a) Rate control<br>b) Rhythm control<br><br>ROWS:<br>1. Always (76–100% of patients)<br>2. Often (51–75%)<br>3. Sometimes (26–50%)<br>4. Rarely (1–25%)<br>5. Never (0%)
C2b	Of the beta-blockers listed, please rank the <u>top three</u> that you use for rhythm control and rate control?<br>*Please rank your top three with 1 being most preferred.*	COLUMNS<br>1. Rhythm control<br>2. Rate control<br><br>ROWS:<br>Beta-blockers:<br><br>1. Acebutolol<br>2. Atenolol<br>3. Betaxolol<br>4. Bisoprolol<br>5. Carvedilol<br>6. Labetalol<br>7. Metoprolol succinate<br>8. Metoprolol tartrate<br>9. Nadolol<br>10. Nebivolol

		11. Penbutolol<br>12. Pindolol<br>13. Propranolol<br>14. Timolol
C3a	How often do you use non-dihydropyridine calcium antagonist/channel blocker (CCB) for a) rate control and b) rhythm control?<br>*Please select one per column*	COLUMNS:<br>a) Rate control<br>b) Rhythm control<br><br>ROWS:<br>1. Always (76–100% of patients)<br>2. Often (51–75%)<br>3. Sometimes (26–50%)<br>4. Rarely (1–25%)<br>5. Never (0%)
C3b	Which non-dihydropyridine calcium antagonist/channel blocker (CCB) do you prefer to use?<br>*Please select one*	1. Diltiazem<br>2. Verapamil
C4	How often do you use digitalis glycosides for a) rate control and b) rhythm control?<br>*Please select one per column*	COLUMNS:<br>a) Rate control<br>b) Rhythm control<br><br>ROWS:<br>1. Always (76–100% of patients)<br>2. Often (51–75%)<br>3. Sometimes (26–50%)<br>4. Rarely (1–25%)<br>5. Never (0%)
C5	Of the following sodium channel blockers, which have you used for patients with AF for long-term use in the last 12 months?<br><br>*Please select all that apply*	1. Quinidine<br>2. Propafenone<br>3. Flecainide<br>4. Disopyramide<br>5. Antazoline<br>6. Cibenzoline

		7. Ranolazine<br>8. Other, please specify _______<br>9. None
C6	Of the following potassium or multichannel K channel blockers, which have you used for patients with AF for long term use in the last 12 months?<br><br>*Please select all that apply*	1. Amiodarone<br>2. Dronedarone<br>3. Sotalol<br>4. Other, please specify _______<br>5. None
C7a and b	Which drug combinations for rhythm control do you use most often, if any?<br>*Please select all that apply*<br><br>For, each combination, please specify which drugs you most commonly use:<br>*Please use the drop-down menus*	Category (multi select):<br>1. AAD + beta blocker<br>2. AAD + calcium channel blocker (CCB)<br>3. AAD + digitalis<br>4. Combinations of AADs<br>5. Other combination<br>6. I do not use drug combinations [exclusive]<br><br>[masked from items selected at above]<br>For, each combination, please specify which drugs you most commonly use:<br>1. AAD + beta blocker, please specify: _____ + ______<br>2. AAD + CCB, please specify: _____ + ______<br>3. AAD + digitalis, please specify: _____ + ______<br>4. Combinations of AADs, please specify: _____ + ______<br>5. Other combination, please specify: _____ + ______
C8	In what percentage (%) of your AF patients overall do you use drug combinations?<br>*Please estimate the % for each of the AF patient subtypes*	1. First onset AF (unclassified)<br>2. Paroxysmal AF<br>3. Persistent AF<br>4. Mixed paroxysmal and persistent<br>5. Long-standing persistent AF<br>6. Permanent AF
C9	In patients with AF on an AAD who experience a recurrence, in what percentage do you:<br><br>*Please type in % per row*	1. Try another AAD (switch)<br>2. Try combinations of AADs (add-on)<br>3. Move to ablation<br>4. Other, please specify

C10	Now we will focus on your use of AADs in patients with AF in four different patient types.<br><br>1. Thinking of patients with AF who have no or minimal structural heart disease…<br><br>Which AAD drug do you most commonly use in each of these patient sub-groups?<br><br>*Select one drug per column*	COLUMNS:<br>1. First onset AF (unclassified)<br>2. Paroxysmal AF<br>3. Persistent AF<br>4. Mixed paroxysmal and persistent<br>5. Long-standing persistent AF<br>6. Permanent AF<br><br>ROWS: (short drug list)<br>1. Amiodarone<br>2. Dronedarone<br>3. Flecainide<br>4. Propafenone<br>5. Sotalol<br>6. Dofetilide (US only)<br>7. Other AAD, please specify ______<br>None
C11	2. <u>For patients with AF who have coronary artery disease...</u><br><br>Which AAD drug do you most commonly use in each of these patient sub-groups?<br><br>*Select one drug per column*	Same options as C10
C12	3. <u>For patients with AF who have heart failure…</u><br><br>Which AAD drug do you most commonly use in each of these patient sub-groups?<br><br>*Select one drug per column*	Same options as C10
C13	4. <u>For patients with AF who have left ventricular hypertrophy (>1.4 cm)…</u><br><br>Which AAD drug do you most commonly use in each of these patient sub-groups?	Same options as C10

	Select one drug per column	
C14	In what % of your patients with paroxysmal or persistent AF do you use the "pill-in-the-pocket approach", as opposed to a daily AAD regimen?<br>*Type in %*	COLUMNS:<br>1. Paroxysmal AF<br>2. Persistent AF<br><br>ROWS:<br>1. Minimal or no heart disease<br>2. Structural heart disease
C15	When you use "pill-in-the-pocket", do you:<br>*Please select one*	1. Use it without rate control<br>2. Use it only in patients taking regular rate control therapy<br>3. Add rate control medication to the "pill-in-the-pocket" therapy
C16	Which rate control therapy do you prefer to use with "pill-in-the-pocket" therapy?<br>*Please select one*	1. Beta-blockers<br>2. CCBs<br>3. Digitalis glycosides
C17	Which AAD drug(s) do you use for the "pill-in-the-pocket" approach?<br>*Please select all that apply*	COLUMNS:<br>1. Minimal or no heart disease<br>2. Structural heart disease<br><br>ROWS: (short drug list)<br>1. Amiodarone<br>2. Dronedarone<br>3. Flecainide<br>4. Propafenone<br>5. Sotalol<br>6. Dofetilide (US only)<br>7. Other, please specify ___
C18	What arrhythmia frequency seems appropriate to you to use the "pill-in-the-pocket" approach?<br>*Please select one*	1. About once a month or more<br>2. Once every 2–3 months<br>3. Every 4–6 months<br>4. Every 7–12 months<br>5. Yearly or more
C19	What investigations do you request routinely (at least yearly) in your patients who are taking each of the following AADs?	COLUMNS:<br>1. Amiodarone<br>2. Dronedarone

	Please select all that apply	3. Flecainide<br>4. Propafenone<br>5. Sotalol<br>6. Dofetilide (US only)<br><br>ROWS:<br>1. ECG<br>2. Renal function<br>3. Electrolytes<br>4. Hepatic function<br>5. Echocardiogram<br>6. Plasma concentration<br>7. Chest x-ray<br>8. Stress (exercise) test/assessment heart rate control<br>9. Thyroid function<br>10. Respiratory function<br>11. Visual/ophthalmology<br>12. Other, please specify _____<br>13. No routine investigations
C20	What, if any, are the main reasons in general for not using the following AADs, in your opinion?<br>*Please select all that apply*<br><br>*Please note, do not report any individual patient experience encountered while being treated with a product*	COLUMNS:<br>1. Amiodarone<br>2. Dronedarone<br>3. Flecainide<br>4. Propafenone<br>5. Sotalol<br>6. Dofetilide (US only)<br><br>ROWS:<br>1. Poor efficacy<br>2. Increased mortality<br>3. Ventricular proarrhythmic effects<br>4. Aggravation of heart failure<br>5. Other side effects<br>6. Specific comorbidity, please specify _______<br>7. Poor general health status of patient

<table>
<tr><td></td><td></td><td>8. Specific patient characteristic, please specify: ________
9. Other, please specify ______
10. None</td></tr>
<tr><td>C21a, b, c</td><td>In your opinion, which of the following safety concerns would you associate with these AADs, if any?

Please select all that apply</td><td>COLUMNS:
1. Amiodarone
2. Dronedarone
3. Flecainide
4. Propafenone
5. Sotalol
6. Dofetilide (US only)

ROWS:
1. Mortality
2. Heart failure
3. Ventricular proarrhythmia
4. Atrial flutter with 1:1 conduction
5. Systemic toxicity (e.g. liver, lung, renal etc.)
6. Bradycardia/conduction system disease
7. No safety risks</td></tr>
<tr><td>C22</td><td>In your opinion, for patients with recurrent AF treated with the following AADs, what is an approximate estimate of drug withdrawal rates at long term (2 years) for safety reasons or side effects in general?

Please think hypothetically</td><td><table>
<tr><td></td><td>0%</td><td>1–2%</td><td>3–10%</td><td>11–25%</td><td>26–40%</td><td>>40%</td></tr>
<tr><td>Amiodarone</td><td></td><td></td><td></td><td></td><td></td><td></td></tr>
<tr><td>Dronedarone</td><td></td><td></td><td></td><td></td><td></td><td></td></tr>
<tr><td>Flecainide</td><td></td><td></td><td></td><td></td><td></td><td></td></tr>
<tr><td>Propafenone</td><td></td><td></td><td></td><td></td><td></td><td></td></tr>
<tr><td>Sotalol</td><td></td><td></td><td></td><td></td><td></td><td></td></tr>
<tr><td>Dofetilide (US only)</td><td></td><td></td><td></td><td></td><td></td><td></td></tr>
</table></td></tr>
<tr><td>C23</td><td>In your opinion, for patients with recurrent AF treated with the following AADs, what is an approximate estimate of drug withdrawal rates at long term (2 years) for efficacy reasons (i.e. lack of a satisfactory clinical effect, even if no complete efficacy)?</td><td><table>
<tr><td></td><td>0%</td><td>1–5%</td><td>6–15%</td><td>16–30%</td><td>31–50%</td><td>>50%</td></tr>
<tr><td>Amiodarone</td><td></td><td></td><td></td><td></td><td></td><td></td></tr>
<tr><td>Dronedarone</td><td></td><td></td><td></td><td></td><td></td><td></td></tr>
<tr><td>Flecainide</td><td></td><td></td><td></td><td></td><td></td><td></td></tr>
<tr><td>Propafenone</td><td></td><td></td><td></td><td></td><td></td><td></td></tr>
<tr><td>Sotalol</td><td></td><td></td><td></td><td></td><td></td><td></td></tr>
</table></td></tr>
</table>

	Please think hypothetically	Dofetilide (USA only) | | | | | |
C24	Do you have concerns using any of the following drugs with:<br>- Apixaban<br>- Dabigatran<br>- Edoxaban<br>- Rivaroxaban<br>- Vitamin K antagonist e.g. warfarin<br><br>*Select all that apply for each column*	COLUMNS (AADs):<br>1. Amiodarone<br>2. Dronedarone<br>3. Flecainide<br>4. Propafenone<br>5. Sotalol<br>6. Dofetilide (US only)<br>7. Beta blockers<br>8. Non-dihydropyridine calcium antagonist/channel blocker (CCB)<br>9. Digitalis<br><br>ROWS:<br>1. Apixaban<br>2. Dabigatran<br>3. Edoxaban<br>4. Rivaroxaban<br>5. Vitamin K antagonists e.g. warfarin and phenprocoumon<br>6. I don’t have any concerns
**Section D: Ablation**		
**Information**	**Questions designed to focus on the use/recommendation of ablation procedures.**	
D1: alternative wording was used dependent on specialty	Now we will focus on your use of ablation as first procedure (de novo) for rhythm control.<br><br>Which ablation procedure do you most commonly recommend in each of these patient sub-groups?<br><br>*Single select*	COLUMNS:<br>1. Paroxysmal AF<br>2. Persistent AF<br>3. Long-standing persistent AF<br>4. Permanent AF<br><br>ROWS:<br>1. PVI alone<br>2. PVI plus other additional ablation lesions

		Cardiologists only: show “Don’t know”
D2	Which patient types are you more likely to refer for ablation, rather than initiation of AAD drug treatment?<br><br>*Please put the options into rank order, where 1=most likely*<br>*If you are not likely to refer these patients for ablation, do not rank them*	1. Subclinical AF<br>2. Asymptomatic recurrent AF<br>3. Mildly symptomatic but infrequent paroxysmal AF<br>4. Frequent symptomatic paroxysmal AF<br>5. Infrequent persistent AF<br>6. Persistent AF (2 or more cardioversions in the past year)<br>7. Long-standing persistent AF (a year or longer)<br>8. Recurrence of AF post-ablation<br>9. Other, please specify____
D3	Are there any patient characteristics that would preclude attempts at ablation?<br><br>*Please select all that apply*	1. Over a specific age, specify _____<br>2. Specific comorbidities<br>3. Left atrial diameter, please specify mm____<br>4. Left ventricular impairment<br>5. Other, specify ____<br>6. None of the above
D4	What percentage of your ablation patients have previously tried an AAD?<br>*Type in % for each row*	1. ____% have not previously tried any AAD<br>2. ____% have previously tried one AAD<br>3. ____% have previously tried more than one AAD<br>4. ____% don’t know
D5	In what % of your patients in the following groups do you use an AAD after the ablation procedure:<br><br>*Type in % per row*	1. Directly after the ablation procedure in all patients irrespective of symptoms/recurrences until first post-ablation visit after 3–6 months ___%<br>2. Directly after ablation procedure in all patients irrespective of symptoms/recurrences for 1–2 months post-ablation ___%<br>3. Any time post-ablation if symptomatic AF recurrences ___%<br>4. Short term if AF recurrence and a re-ablation is planned ___%<br>5. Long term if AF recurrence and a re-ablation is not planned ___%
D6ai<br><br>D6aii	1. <u>Thinking about your patients with paroxysmal AF:</u><br><br>Which AAD drugs do you tend to use in patients after ablation at the following time points:	COLUMNS:<br>1. Directly after the ablation procedure in all patients irrespective of symptoms/recurrences until first post-ablation visit after 3–6 months

	- Directly after the ablation procedure in all patients irrespective of symptoms/recurrences until first post-ablation visit after 3–6 months<br>- Directly after ablation procedure in all patients irrespective of symptoms/recurrences for 1–2 months post-ablation<br>- Any time post-ablation if symptomatic AF recurrences<br>- Short term if AF recurrence and a re-ablation is planned<br>- Long term if AF recurrence and a re-ablation is not planned<br><br>*Select one drug per column*	2. Directly after ablation procedure in all patients irrespective of symptoms/recurrences for 1–2 months post-ablation<br>3. Any time post-ablation if symptomatic AF recurrences<br>4. Short term if AF recurrence and a re-ablation is planned<br>5. Long term if AF recurrence and a re-ablation is not planned<br><br>ROWS:<br>1. Amiodarone<br>2. Dronedarone<br>3. Flecainide<br>4. Propafenone<br>5. Sotalol<br>6. Dofetilide (US only)<br>7. Other AAD, please specify _____<br>8. No drug treatment<br><br>D6aii: FOR EACH TIME POINT, TICK BOX:<br>Is this drug used for recurrence or prophylactically?<br><br>a) For recurrence<br>b) Prophylactically<br>c) No drug treatment
D6bi<br><br>D6bii	2. <u>Thinking about your patients with non-paroxysmal AF:</u><br><br>Which AAD drug do you tend to use in patients after ablation at the following time points?<br><br>1. Directly after the ablation procedure in all patients irrespective of symptoms/recurrences until first post-ablation visit after 3–6 months	COLUMNS:<br>1. Directly after the ablation procedure in all patients irrespective of symptoms/recurrences until first post-ablation visit after 3–6 months<br>2. Directly after ablation procedure in all patients irrespective of symptoms/recurrences for 1–2 months post-ablation<br>3. Any time post-ablation if symptomatic AF recurrences<br>4. Short term if AF recurrence and a re-ablation is planned<br>5. Long term if AF recurrence and a re-ablation is not planned

	2. Directly after ablation procedure in all patients irrespective of symptoms/recurrences for 1–2 months post-ablation<br>3. Any time post-ablation if symptomatic AF recurrences<br>4. Short term if AF recurrence and a re-ablation is planned<br>5. Long term if AF recurrence and a re-ablation is not planned<br><br>*Select one drug per column*	ROWS:<br>1. Amiodarone<br>2. Dronedarone<br>3. Flecainide<br>4. Propafenone<br>5. Sotalol<br>6. Dofetilide (US only)<br>7. Other AAD, please specify ____<br>8. No drug treatment<br><br>D6bii - FOR EACH TIME POINT, TICK BOX:<br>Is this drug used for recurrence or prophylactically?<br><br>a) For recurrence<br>b) Prophylactically<br>c) No drug treatment
D7	Which AAD do you generally use in a hypothetical patient who has an atrial tachyarrhythmia directly after the ablation procedure?<br><br>*Select one drug per column*	COLUMNS: Arrhythmias seen after the ablation procedure (not the primary ablated arrhythmia)<br>1. Paroxysmal AF<br>2. Persistent AF<br>3. Atrial tachycardia/atypical flutter<br>4. Common atrial flutter<br><br>ROWS:<br>1. Amiodarone<br>2. Dronedarone<br>3. Flecainide<br>4. Propafenone<br>5. Sotalol<br>6. Dofetilide (US only)<br>7. Other AAD, please specify<br>8. No AAD drug treatment<br><br>D7i - FOR EACH TIME POINT, TICK BOX:

		Is this drug used for recurrence or prophylactically?<br><br>a) For recurrence<br>b) Prophylactically<br>c) No drug treatment
D8	In general, for those patients who receive an AAD directly after the ablation procedure (i.e. within first 3-6 months), do you tend to use a new AAD or one that the patient previously received prior to ablation?<br><br>*Select one option*	1. AAD that was unsuccessful prior to ablation<br>2. AAD that was partially successful following first ablation<br>3. AAD that was not used before<br>4. Drug combination that was not used before<br>5. Rate controlling drug<br>6. Other, please specify ______
D9	Does the energy source (cryo or RFA) influence AAD therapy after PVI?<br>*Select one option*	1. Yes, with cryo I use _____<br>2. Yes, with RFA I use _____<br>3. No<br>4. Don't know
D10	How do you judge the efficacy of ablation?<br>*Please select all that apply*	1. Recurrence of any atrial fibrillation irrespective of duration or associated symptoms<br>2. Single symptomatic AF/atrial tachycardia<br>3. High burden of AF<br>4. Need for hospitalization<br>5. Other, specify ______
D11	What percentage of your patients referred for ablation have a clinically significant recurrence that mandates a re-ablation within 1 year?<br><br>*Please type % for each column*	ROW:<br>1. Paroxysmal AF<br>2. Persistent AF<br>3. Long-standing persistent /permanent AF<br><br>COLUMN:<br>__% patients who undergo re-ablation
D12	And in patients who receive an AAD after ablation, do you tend to use a new AAD or one that the patient previously received prior to ablation, or is rate control sufficient?<br>*Select one option*	1. AAD that was unsuccessful prior to ablation<br>2. AAD that was partially successful following first ablation<br>3. AAD that was not used before<br>4. Drug combination that was not used before<br>5. Rate controlling drug<br>6. Other, please specify ______

Section E: Patient types/scenarios		
**Information**	**Questions based on several different AF patient profiles, allowing physicians to consider how they would treat these patients. Physicians were encouraged to draw on experiences with real patients where possible.**	
E1a-j	In a patient with recurrent symptomatic AF in whom AF ablation is deferred or not planned, what is your first pharmacological option with AAD if the hypothetical patient …<br><br>a. ...has no or minimal signs for structural heart disease (i.e. no left ventricular hypertrophy nor LV dilatation, and no ischemic heart disease)?<br><br>b. …has history of coronary artery disease (MI 5 years ago, no active ischemia), normal left ventricular EF, with no current signs/symptoms of ischemia?<br><br>c. …has history of mild stable heart failure, NYHA II, LVEF 45%, no hospitalization during the least two years?<br><br>d. …has mild left ventricular hypertrophy (<14 mm LV thickness at echocardiogram)?<br><br>e. …has hypertensive moderate/severe left ventricular hypertrophy (≥14mm LV thickness at echocardiogram)?<br><br>f. …has heart failure with preserved ejection fraction (>50%)?<br><br>g. … has major comorbidities but without severe heart failure?	[short DRUG LIST] plus beta blockers<br>And other and none

	h. …is an asymptomatic patient with evidence of CAD on a cardiac CT scan, but no IHD history and a negative stress test?<br><br>i. …has bradycardia tendency or intraventricular conduction defects?<br><br>j. …has paroxysmal AF and sinus node dysfunction?<br><br>k. …has moderate chronic kidney disease (eGFR 30–60 ml/min/1.73m^2)?<br><br>l. … has severe chronic kidney disease (<30 ml/min/1.73m^2)<br><br>m. … has hypertrophic cardiomyopathy and AF?	
E2	What are the general differences in how you treat men versus women?<br>*Please select all that apply, and explain your response(s)*	1. Rate control vs rhythm control, please explain ________<br>2. Ablation vs AADs, please explain ________<br>3. Choice of AAD, please explain ________<br>4. Choice of AAD dose, please explain ________<br>5. I don't treat men and women differently
E3	What is your age limit for rhythm control with drugs, if any?<br>*Select one option*	1. >65 years of age<br>2. >70 years of age<br>3. >75 years of age<br>4. >80 years of age<br>5. No limit
E4	What is your age limit for rhythm control with ablation, if any?<br>*Select one option*	1. >65 years of age<br>2. >70 years of age<br>3. >75 years of age<br>4. >80 years of age<br>5. No limit
**Section F: Future AF landscape**		
**Information**	**Questions designed to investigate physician opinions on the future for management of patients with AF**	

F1a	Thinking ahead…<br><br>Do you think the uptake of first-line ablation will change in the next 3–5 years?<br>*Select one option*	1. No change<br>2. Decrease<br>3. Increase<br>4. Don’t know
F1b	You stated the uptake of ablation will decrease in the next 3–5 years. Please tell us the approximate decrease.<br>*Please select one*	1. 10%<br>2. 25%<br>3. 50%<br>4. >50%
F1c	You stated the uptake of ablation will increase in the next 3–5 years. Please tell us the approximate increase.<br>*Please select one*	5. 10%<br>6. 25%<br>7. 50%<br>8. >50%
F2	Thinking about the new AADs that will be coming to market in the next 5–10 years, what changes or improvements in AADs would you LIKE to see, ideally?<br><br>*Please rank these in terms of what you would like to see*	1. Greater antiarrhythmic efficacy<br>2. Drugs that reverse remodelling<br>3. Less proarrhythmia<br>4. Less effect on ventricular function<br>5. Fewer complications<br>6. New modes of action<br>7. Other, please specify _________
F3	Where do you see as the most important indications/situations for AADs in the future?<br><br>*Select top 3*	1. For prevention of AF recurrence<br>2. For prevention of post-ablation AF recurrence<br>3. Whilst waiting for an ablation<br>4. For ablation failure or as hybrid therapy<br>5. For patients not willing or with high risk of ablation<br>6. For patients due to health care limitations<br>7. For patients unable to afford an ablation<br>8. Other, please specify _________<br>9. No place for AADs in the future [exclusive]
F4	In your opinion, is there a need for clinical trials of AADs post-ablation that were ineffective prior to ablation?<br>*Select one option*	1. Yes<br>2. No<br>3. Not sure
F5	Did the recent 2020 ESC Guidelines on AF influence your responses to this survey?	1. Yes

		2. No<br>3. Not sure<br>(if yes/no): Please explain: ________
F6a	Are you aware of the EAST study presented at the European Society of Cardiology 2020 congress?	1. Yes<br>2. No<br>3. Not sure
F6b and c (2 questions on 1 page)	a) Did the results of the EAST study influence your choice between rate and rhythm control?<br>b) Has it influenced the choice of AAD versus ablation?	1. Yes<br>2. No<br>3. Not sure<br>(if yes/no): Please explain: ________
